# Controllable Sliding Transfer of Wafer‐Size Graphene

**DOI:** 10.1002/advs.201600006

**Published:** 2016-03-15

**Authors:** Wenjing Lu, Mengqi Zeng, Xuesong Li, Jiao Wang, Lifang Tan, Miaomiao Shao, Jiangli Han, Sheng Wang, Shuanglin Yue, Tao Zhang, Xuebo Hu, Rafael G. Mendes, Mark H. Rümmeli, Lianmao Peng, Zhongfan Liu, Lei Fu

**Affiliations:** ^1^ College of Chemistry and Molecular ScienceWuhan UniversityWuhan430072P. R. China; ^2^ State Key Laboratory of Electronic Thin Films and Integrated DevicesUniversity of Electronic Science and Technology of ChinaChengdu610054P. R. China; ^3^ Department of ElectronicsPeking UniversityBeijing100871P. R. China; ^4^ IFW DresdenP. O. Box 270116Dresden01069Germany; ^5^ Center for NanochemistryCollege of Chemistry and Molecular EngineeringPeking UniversityBeijing100871P. R. China

**Keywords:** graphene, liquid metal, sliding transfer, ultrafast, wafer size

## Abstract

**The innovative design of sliding transfer** based on a liquid substrate can succinctly transfer high‐quality, wafer‐size, and contamination‐free graphene within a few seconds. Moreover, it can be extended to transfer other 2D materials. The efficient sliding transfer approach can obtain high‐quality and large‐area graphene for fundamental research and industrial applications.

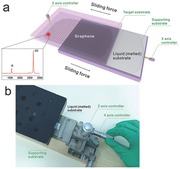

The successful isolation of high‐quality single‐layer or few‐layer graphene by mechanical exfoliation has unleashed a flurry of research activities in 2D carbon worldwide over the past few years.^1^, ^2^, ^3^, ^4^, ^5^ The ease of production and low cost make exfoliation of graphite the most popular route to prepare graphene whenever and wherever possible.^6^, ^7^, ^8^ However, this generally involves using either an adhesive tape to attach to the surface of graphite and using force to vertically peel off the tape plus graphene layers attached^1^, ^7^, ^8^ or by rubbing the surface of graphite against another material to slide off graphene sheets from the bulk.^6^, ^9^ Thus, this technique fails to provide sufficient output yield, large size and layer‐number homogeneity for many applications owing to multiple cleavage planes and strong interlayer van der Waals interactions in the normal direction.^8^ Moreover, the produced material often contains exfoliating agent remnants such as glues due to the pre‐press to obtain compact contact and the following violent peeling to achieve separation, and micromechanical action may induce strains on the graphene layer during adherence on a substrate and introduce various types of defects, including atomic vacancies, wrinkles or cracks, and microscopic corrugation. These inhomogeneities and defects may lower the electrical performance of graphene devices since they break translational or rotational symmetry.^10^, ^11^ Some studies have been conducted to improve the controllability of the exfoliation process. Instead of adhesive tape which relies on enforced press to achieve full contact, a zinc layer was implemented over the multilayer flakes of graphite to realize better contact via refined sputtering process and was subsequently mildly removed in diluted hydrochloric acid, which resulted in the controllable removal of a single atomic layer of carbon material, one layer at a time.^12^ The procedure can be repeated to remove additional carbon layers, nevertheless, this improved transfer method can be quite cumbersome and still fails to achieve the whole uniformity of the as‐obtained graphene. In conclusion, the core problem lies in the strong layer interaction of exfoliated layers and the improper mode to apply force, which hampers the controllability of graphene transferred onto target substrates. Accordingly, we attempt to design an innovative transfer method based on uniform graphene on an easily exfoliated support via applying a sliding force parallel to the interfacial shear slip, which not only inherits the simplicity of traditional mechanical cleavage but also improves the interface contact and lowers the exfoliation energy, thus improves its controllability of uniformity and size.

Here, we develop a novel sliding transfer strategy, which is inspired by the natural phenomenon of a snail secreting mucus (Figure S1, Supporting Information).^13^ In a similar fashion, given a unique original substrate (OS) and an appropriate target substrate (TS), when the OS which bears graphene parallelly “crawls” on the TS, the graphene will closely adhere to the TS due to the easy separation of the OS and the lateral push force resulted from the horizontal movement. Thus, an OS which owns certain fluidity will facilitate the sliding transfer process of graphene. A kind of specific original substrates–liquid metals, such as gallium (Ga), indium (In), and their alloys, which have proved to be outstanding catalytic substrates for strictly monolayer and highly uniform graphene growth come to mind.^14^, ^15^ In principle, once the metal substrates supporting the growth of graphene are melted, such a sliding transfer is applicable.

**Figure** **1**a schematically exhibits the sliding process of graphene with accurate control both in the spatial displacement and the exerted sliding force. In a state that graphene on the original liquid (melted) substrate was faced with the target substrate, the contact of graphene and the TS can be adjusted with a Z axis controller, and the horizontal sliding force can be exerted by an X axis controller. When the original liquid Ga substrate was slided to the right, graphene completely adhered to the target substrate. A tiny amount of Ga can be easily removed only via rinsing with diluted hydrochloric acid thoroughly.^14^ The inset of a Raman spectrum demonstrates the successful transfer, which is corresponding to the region of graphene irradiated by a laser spot, where the intensity of the 2D peak at 2677 cm^–1^ with a full width at half maximum (FWHM) of 27.0 cm^–1^ is more than four times the intensity of the G peak at 1582 cm^–1^. The D peak, indicating defects of graphene, is not obvious, which attests to extremely high crystalline quality. The equipment corresponding to Figure 1a is shown in Figure 1b. The detailed transfer information can be seen in Figure S3 of the Supporting Information and the given photograph of a 1 × 1 cm^2^ monolayer graphene transferred onto a 300 nm SiO_2_/Si substrate exhibits excellent uniformity and integrality with a clean surface. The ingenious design of sliding transfer actually can be dated back to an institutional technique—floating glass,^16^ which takes full advantage of ultra‐smooth surface of the liquid and has been applied to the preparation of ultra‐large‐size glass until now. In view of this, the sliding transfer method is expected to achieve large‐size graphene. When employing flexible and transparent ethylene‐vinyl acetate copolymer (EVA) plastic as the target substrate, large‐area and uniform graphene film can be achieved via rolling–sliding process since its relatively better flexibility and smoother surface can facilitate more uniform and compact contact with liquid gallium. What is more, the whole sliding transfer of graphene only takes a few seconds due to the extreme ease of peeling process. Notably, the features of Ga offer access to achieve graphene of 100% monolayer coverage on a support with excellent separability,^14^ which makes it possible to obtain scalable and controllable production of uniform graphene. In our proposed transfer method, only by exerting a sliding force, the graphene floating on the original melted substrate can be transferred onto the target substrate rapidly. The simplicity of the technique and high efficiency with the companion of good controllability makes our proposed transfer method overcome the core difficulties (speedability and simplicity) of the industrial applications of graphene.

**Figure 1:**
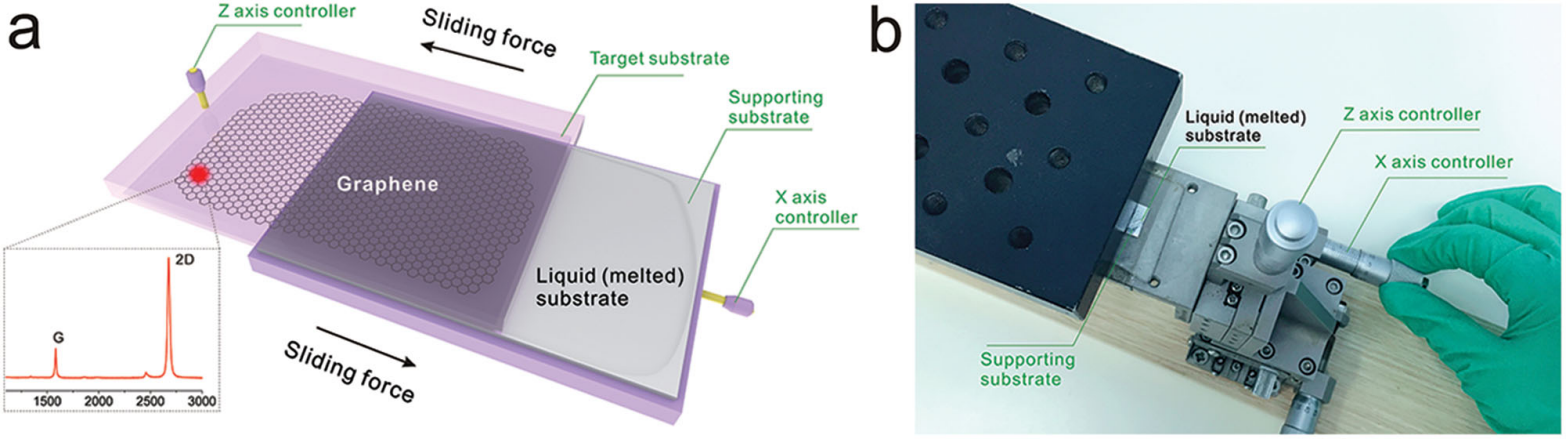
Illustration of the sliding transfer method for obtaining uniform graphene. a) The scheme shows the sliding process. The contact between liquid substrate–graphene and target substrate can be accurately controlled by the Z axis controller, after which the liquid substrate can slide towards the right via rotating the X axis controller. Thus, the graphene was transferred onto the target substrate. The inset of the Raman spectrum demonstrates the successful transfer, which is corresponding to the region of the graphene irradiated by a laser spot. b) A photograph of the equipment for sliding transfer.

The core competence of our sliding method results from the ease of metallic atom delocalization at melted state and the applied sliding force. Any original substrate, once melted, can easily achieve facile atom delocalization, which establishes the foundation for the layer–layer separation of liquid metal and the applied sliding force further promotes such a process. Here we take gallium to illustrate our sliding transfer technique.

Ga has a near room‐temperature melting point of 29.8 °C, which is decided by the strength of the metallic bond that depends on the number of delocalized electrons.^17^ As a result, the metallic bonds of Ga are not strong enough to ensure the atoms to form perfect crystal structures with close packing, for which the big radius of Ga atoms also contribute a lot. Accordingly, the bonding between the two nearest neighbors is more likely covalent.^18^, ^19^ Once melted, the imperfect crystal structures will be broken down and the bonds are loosened. Thus, when at a molten state, a part of Ga will be separated from the rest with some metallic bond broken under a certain stress. Another significant factor of the sliding transfer lies in the horizontal sliding force. First, the adhesion energy (1.5 eV)^20^ of Ga on graphene must be basically matched with the adhesion energy of graphene–TS, which offers a possibility for the tight contact to realize the successful transfer of graphene. Generally, the commonly employed target substrates, such as SiO_2_/Si,^21^ quartz,^22^ and flexible and transparent materials (EVA)^23^ all can be good choices because of the matched adhesion energies. However, only the matched adsorption will not make a successful transfer. Another key factor is the direction of the applied force—horizontal sliding force. When the TS comes into contact with the graphene/Ga via a prepress, a tiny deformation of graphene supported by liquid substrate will occur around the contact interface, as shown in **Figure** **2**a. Upon sliding parallel to the contact interface, as shown in Figure 2b, the deformation will transform to be eased. After a certain sliding distance, the deformation becomes small gradually and reaches at a steady‐state level, resulting in a maximum steady‐state contact area, thus increasing the graphene–TS interaction (Figure 2b).^24^ Owing to the enhanced graphene–TS interaction, graphene is more inclined to attach to the TS, thus achieving a more tight contact between graphene and the TS. Furthermore, the horizontal sliding force makes it easier to overcome the Ga–Ga adhesion and make the Ga layers horizontally staggered. Therefore, the horizontal sliding force enhances ultracontact of the graphene–TS and easily facilitates the separation of Ga layers, promoting to transfer large‐size graphene onto the TS. On the contrary, when under a vertical pulling force, the deformation of graphene supported by the liquid metal will be aggravated due to the fact that the vertical pulling force is in the opposite direction of the strong graphene–Ga and Ga–Ga interactions. Thus, with such a ponderous and inhomogeneous separating mode, graphene fails to achieve good contact with the TS and the separation process will occur unevenly (Figure 2c). The vertically ‐transferred graphene on TS will be diminutive and damaged seriously, which is similar to mechanical exfoliation. Therefore, such a horizontal sliding force generates the key advantages over the vertical pulling force, that is, it enhances interaction of graphene–TS for an ultracontact between graphene and the TS; it enables a more moderate force for overcoming the Ga–Ga adhesion to transfer graphene than the vertical pulling force, and minimizes the impact of Ga–Ga layer separation on graphene, such as strain. To further demonstrate the mechanism mentioned above, we carried out tensile measurements during the sliding process with a dynamometer. The value corresponding to the critical separating state was determined as the effective pull‐off force. Figure 2d exhibits two datasets of 20 vertical force and sliding force values, illustrating that horizontal sliding force is more moderate than vertical pulling force. And the horizontal sliding force is much smaller than the intrinsic strength of graphene, which will not damage the graphene during such a sliding process.^25^ Thus, a moderate horizontal sliding force further create this large‐size and crack‐decreased transfer technique.

**Figure 2:**
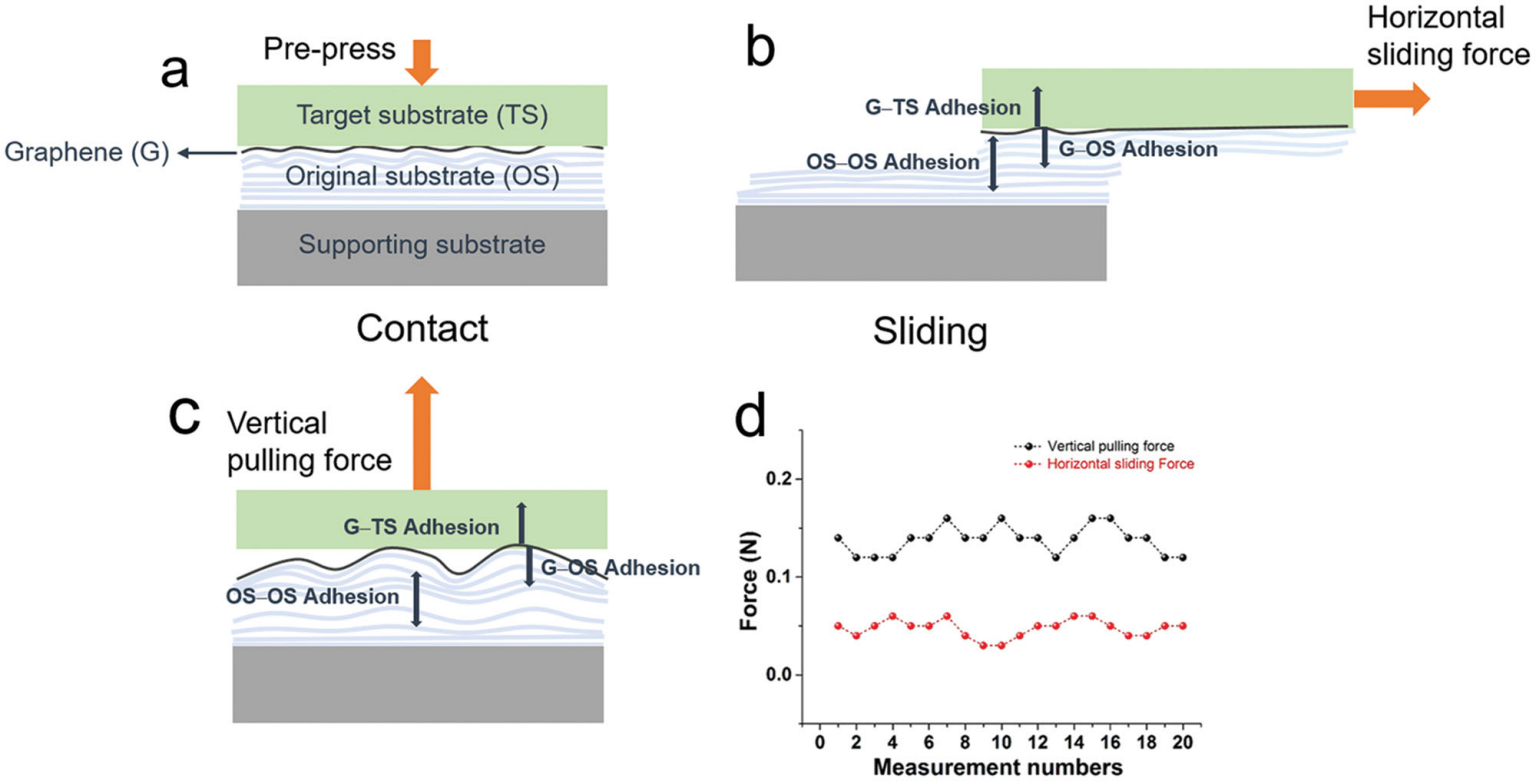
Mechanism of sliding transfer of graphene. a) The initial state in which the TS contacted with graphene/OS supported by the supporting substrate via a prepress process. b) The state of the integrated sample in a horizontal sliding process, in which the G–TS adhesion is basically matched with the G–OS adhesion marked by red unfilled arrows, and the red filled arrow indicates the OS–OS adhesion. Under a smaller horizontal sliding force, the G–TS contact area becomes much larger, and the OS layers are horizontally staggered. c) The state of the integrated sample under a larger vertical pulling force, in which the G–TS contact area becomes smaller. The red arrows represent the same meanings as those in (b). d) The comparison of horizontal sliding force and vertical pulling force for the transfer of graphene.

**Figure** **3** shows the excellent results of the transferred graphene. Figure 3a shows a typical optical image of the sliding transferred graphene on SiO_2_/Si. The graphene film appears to be highly uniform and intact, with no visible defects such as cracks and folds. To further exhibit the whole quality of the transferred graphene,^26^ refined Raman mapping was conducted. The intensity ratios *I*_D_/*I*_G_ and *I*_2D_/*I*_G_ were calculated by integrating the range of the intensity ratio for 256 spectra with mapping for 15 × 15 μm^2^ (Figure 3b,c). The distribution of the *I*_2D_/*I*_G_ intensity ratio is centered at a value of 3.50, which demonstrates that the transferred graphene is monolayer. As for *I*_D_/*I*_G_, the intensity ratio is nearly zero, which confirmed the extremely low defect. Statistical analysis of G peaks shifts by Raman was conducted to show the gentleness of the sliding transfer process, as seen in Figure 3d. The position of G peak distributes over a range of 1578–1581 cm^–1^, which is blue shifted as compared to that from mechanically transferred monolayer graphene on SiO_2_/Si (1580–1588 cm^–1^).^27^ This can be explained by the different strain introduced from the sliding process and the uniform G peak shifts over a large area confirms the gentle and uniform applied force of the sliding technique which has no damage to the transferred graphene. In addition, the excellent uniformity shown in the Raman mapping also exhibits its outstanding advantage over traditional transfer methods. Significantly, the sliding transfer method also makes it possible to directly transfer graphene onto the standard lacey carbon grids instead of traditional complex transmission electron microscopy (TEM) sample preparation. Figure 3e shows a selected area electron diffraction (SAED) image from a relatively large graphene area. To confirm that the observed reflexes are from monolayer graphene and not from AB Bernal stacked graphene, we examined the relative intensity of the inner $\left\{10\bar{1}0\right\}$ and outer $\left\{11\bar{2}0\right\}$ spots. For monolayer, the inner spots are more intense than the outer spots, as we observe (Figure 3f), confirming monolayer graphene. On such a clean graphene film, we further confirm the presence of monolayer graphene by finding holes in the membrane to count the layers (Figure 3g). The single‐layer feature and high crystallinity of the graphene were also supported by the low voltage aberration‐corrected, high resolution transmission electron microscopy (LVAC–HRTEM) (Figure 3h), which highlights the sixfold symmetry single‐crystal nature of the graphene.

**Figure 3:**
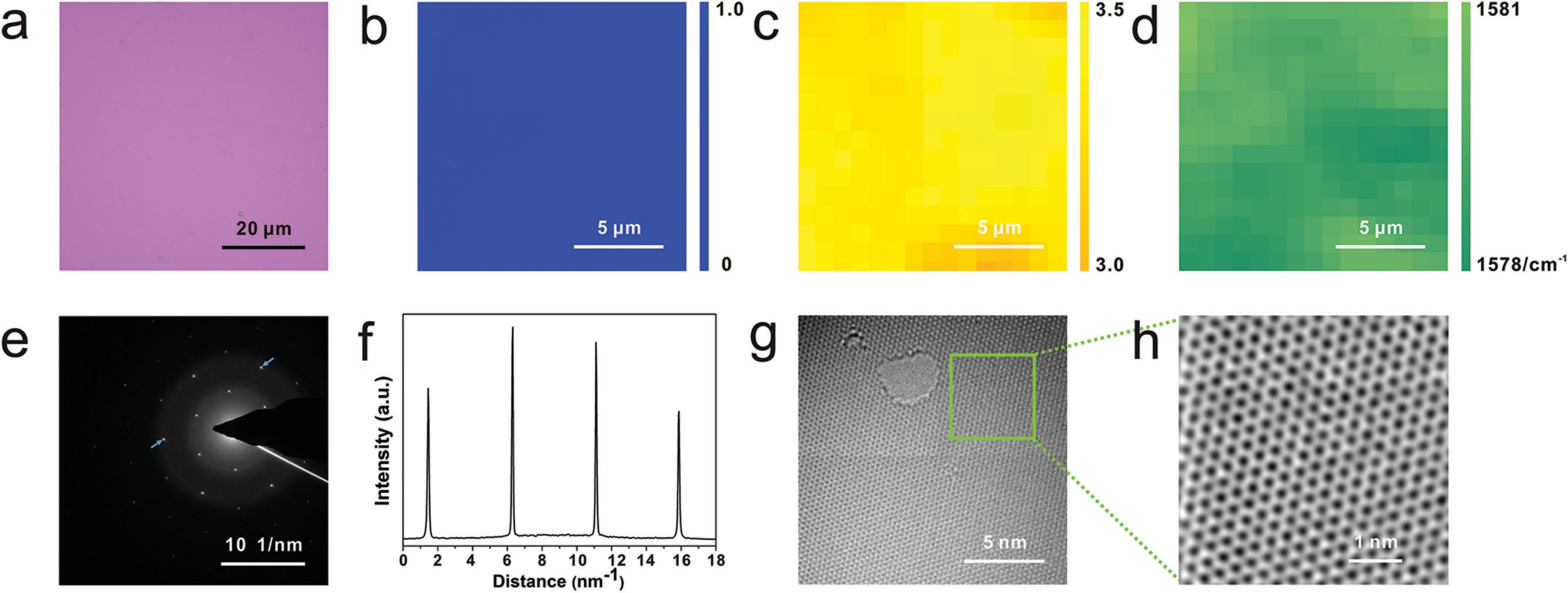
Characterizations of sliding transferred graphene. a) Optical microscope image of graphene transferred onto a SiO_2_/Si substrate through sliding transfer. b) Raman mapping of intensity ratio of 2D peak over G peak of graphene for overlayer analysis. c) Raman mapping of intensity ratio of D peak over G peak of graphene for defect evaluation. d) Raman mapping of G peaks positions of graphene for the analysis of strain distribution. The spatial and spectral resolutions of the measurements are 1 μm and 1 cm^–1^, respectively. e) Selected area electron diffraction pattern from the graphene. f) Intensity profile over the $\left\{10\bar{1}0\right\}$(outer) and $\left\{11\bar{2}0\right\}$ (inner) spots from the SAED pattern (in panel (e)), confirming monolayer graphene. g) HRTEM image of monolayer graphene without contaminants. h) Magnified region of graphene from (e) showing the typical honeycomb lattice of graphene.

X‐ray photoelectron spectroscopy (XPS) was employed to demonstrate that the as‐transferred graphene should be totally metal‐free after washing by diluted HCl. **Figure** **4**a shows the XPS survey spectrum of only signals of C, O, and Si. As shown in Figure 4b, there is no detectable Ga content, which is residue‐free compared with the residues of poly(methyl methacrylate) (PMMA), the solvents or the ferric chloride etchant in the traditional transfer method. Furthermore, the comparisons between sliding transfer and conventional PMMA‐assisted transfer are introduced to declare the super cleanliness, reduced cracks and wrinkles. For traditional PMMA‐assisted transfer, the high surface tension of water and PMMA etching usually exert a pulling force on the graphene, and undesirable ripples, folds or edge curls are created.^27^, ^28^ TEM was employed to further show the ultra‐cleanliness of such a sliding transfer method. As seen in Figure 4c, graphene resting on lacey carbon contains many wrinkles and much pollution. Therefore, intensive efforts are often put into cleaning the graphene sheet, such as annealing in air,^29^ in which the presence of oxygen causes the formation of defects because the graphene sheet is susceptible. While for sliding transfer, such drawbacks can be conquered fundamentally because of no introduction of any pollution sources. Figure 4d shows a large‐area integrated image stitched by many TEM images captured at the lowest magnification, in which there exists no any contaminant, indicating the remarkable superiority of the sliding transfer method over traditional PMMA‐assisted transfer method—cleanliness. Atomic force microscopy (AFM) was also used to demonstrate the cleanliness and microcosmic surface smoothness of the sliding transfer method compared with the traditional PMMA‐assisted transfer. The PMMA‐transferred graphene on SiO_2_/Si in Figure 4e is full of PMMA residues and numerous of wrinkles. However, Figure 4f shows a typical AFM image of the sliding transferred graphene film on SiO_2_/Si, where wrinkles and cracks are hardly visible and no impurities exist in the interface between graphene and the substrate, showing an ultrasmooth film and fully ultraconformal contact with the target substrate. More information about the uniformity, cleanliness, and size of transferred graphene compared with PMMA‐assisted transferred graphene and mechanically transferred graphene were summarized in Figure S6 of the Supporting Information. The sliding transfer indeed owns a clear advantage over the other two methods.

**Figure 4:**
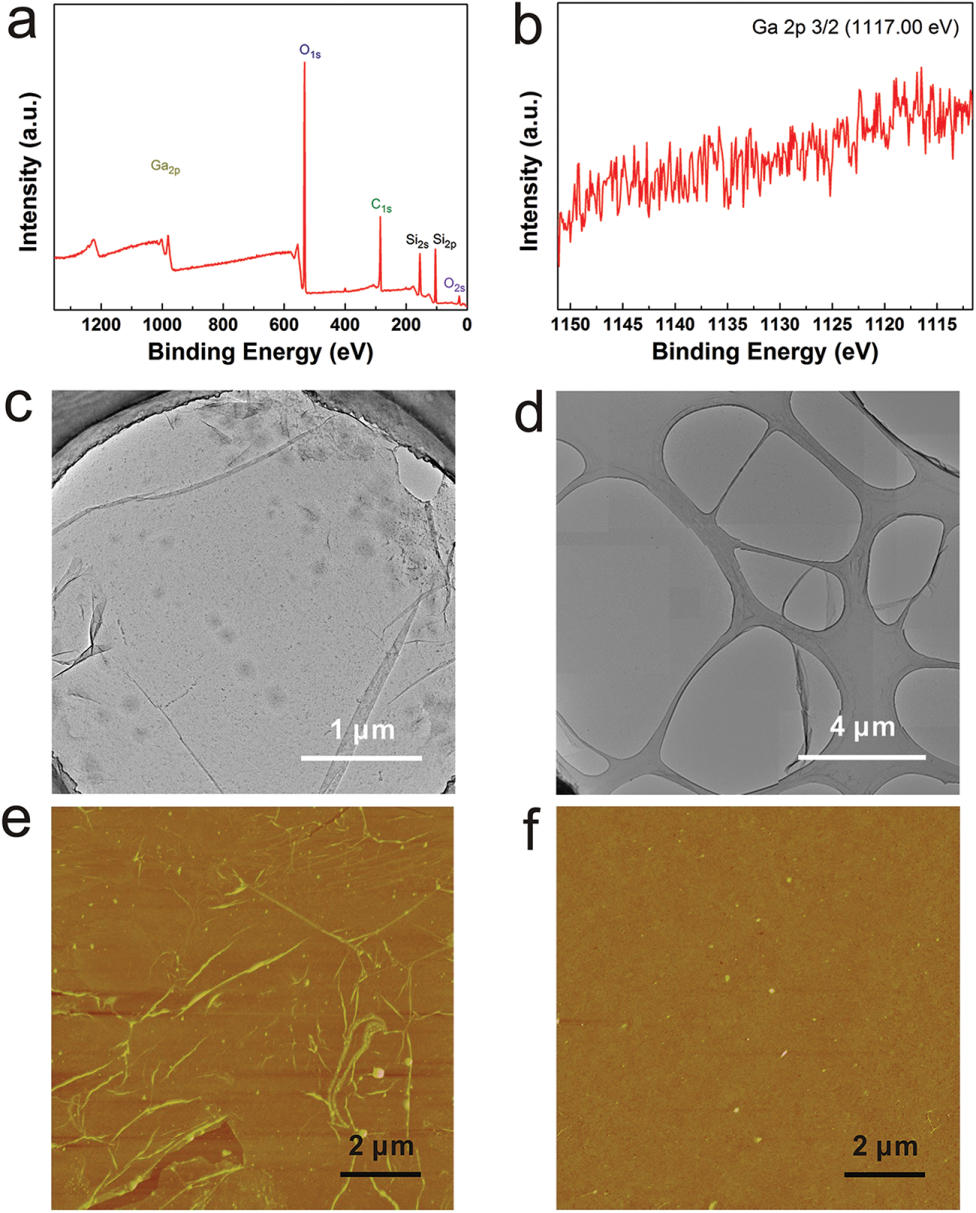
XPS characterization for sliding transferred graphene free from Ga residues and two methods comparisons of TEM sample preparation and AFM characterizations a) XPS survey spectrum of the transferred graphene on SiO_2_/Si. b) High resolution XPS spectrum of Ga 2p. c) TEM image of graphene transferred by traditional PMMA‐assisted method. d) TEM image of sliding transferred graphene resting on lacey carbon at low magnification. e) AFM image of PMMA‐assisted transferred graphene on SiO_2_/Si. f) AFM image of sliding transferred graphene on SiO_2_/Si to show more details about surface morphology.

We then studied the universality the novel transfer method, and transparent quartz and an emerging 2D material–h‐BN were adopted as examples. The clean scanning electron microscopy (SEM) image and Raman spectrum in Figure S4a,c of the Supporting Information present a defect‐free and single‐layer graphene on quartz. In addition, Figure S4b,d of the Supporting Information also demonstrated the successful transfer of h‐BN by the sliding transfer. These results manifest that the sliding transfer process can be directly extended to other substrates and other 2D materials, which is important for a number of applications of electronic and optical devices. On the other hand, the original support in such a sliding transfer strategy can also be altered to meet various demands. Ga–In (76%–24%) alloy of which the melting point is as low as 16 °C and Ga–In–Sn (62%–25%–13%) of which the melting point is as low as 5 °C,^30^ also exhibit good catalytic activity for monolayer graphene growth and have been selected as an available support that can be applied to sliding transfer. The transferred graphene also owns high‐quality and good uniformity, which makes lower‐temperature sliding transfer possible. As shown in Figure S5 of the Supporting Information, clean and uniform graphene films on SiO_2_/Si substrates were obtained by sliding transfer of graphene floating on Ga–In and Ga–In–Sn and the Raman spectra were the feature of single‐layer graphene.

It is worth mentioning that our sliding transfer method can also transfer large‐area graphene onto the flexible and transparent EVA plastic, which is similar to the roll‐to‐roll transfer. The transfer process is shown in Figure S7 of the Supporting Information. **Figure** **5** shows the characterizations of large‐area graphene on EVA plastic via the rolling–sliding process. Figure 5a shows a typical Raman spectrum of transferred graphene on EVA plastic, in which a peak at around 2678 cm^–1^ on EVA plastic can be distinctly observed, and it is ascribed to the 2D peak of the graphene. In Figure 5b,c, Raman spectroscopy mappings over two large‐area regions of 1 × 1 cm^2^ were conducted, which were corresponding with the locations at the left and the right regions of graphene on EVA plastic as marked in Figure 5e (acquisition step = 1 mm). The color maps of 2D peak positions of graphene from the dotted areas highlight the full coverage and high uniformity of the transferred graphene over EVA plastic. Then the optical and electrical properties and integrality of graphene/EVA were evaluated. The optical transmittance of graphene on EVA is found to be ≈95.5% at visible region (Figure 5d), corresponding to monolayer graphene. Note that the absorption of substrate is taken off. Moreover, the sheet resistance of graphene/EVA is measured as 3124 Ω sq^−1^, which is much lower than the value (≈5200 Ω sq^−1^) in the previous report.^23^ In addition, Figure 5e demonstrates the electrical conductivity of graphene transferred onto EVA plastic. The whole transparent EVA plastic film covered with graphene was used as a conductive medium in the whole circuit. The LED bulbs were lightened up when the two metal pens simultaneously contact with the graphene film. With one metal pen fixed on one end of the film and the other pen kept intermittent contact with the other end of the film, the LED bulbs will be lightened up on and off. The considerably good optical and electrical properties of graphene/EVA film enable its potential application in transparent electronic device.

**Figure 5:**
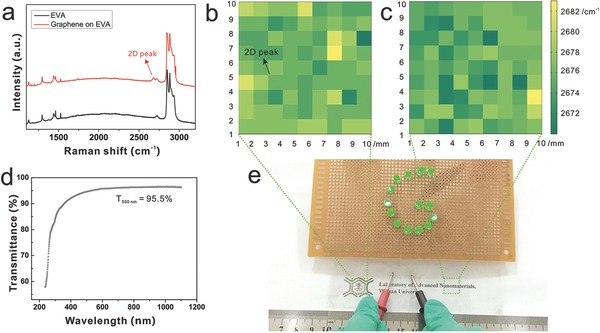
Characterizations of rolling–sliding transfer for large‐area graphene onto flexible and transparent EVA plastic. a) A typical Raman spectrum of graphene on EVA plastic. b,c) Colormaps derived from Raman mapping results acquired from the dotted areas in (e). d) Typical UV–vis–NIR spectrum of graphene/EVA film. e) A photograph of clean and large‐area graphene transferred onto EVA plastic and a simple LED circuit in which the transferred graphene acts as a part of wire for the closed circuit.

To investigate the electrical quality of the sliding transferred graphene onto SiO_2_/Si, back‐gate field effect transistors were fabricated on 300 nm SiO_2_/p‐Si substrates using conventional electron beam lithography. The source and drain electrodes were deposited using Ti/Pd/Au (0.5/30/20 nm) with a separation of 5 μm (Figure S8a, Supporting Information). Figure S8b of the Supporting Information demonstrates the transport characteristics (*I*_ds_–*V*_g_) of the field effect transistor that was measured under ambient conditions. The V‐shaped ambipolar property is typical for single‐layer graphene with a zero bandgap. The extracted carrier mobility of electrons for this device is 3625 cm^2^ V^–1^ s^–1^, which is comparable to the average value of graphene at current condition. The slightly positive Dirac point indicates that the transferred graphene is weakly p‐type doped. Moreover, for the better comparison of the electrical property, we also measured the carrier mobility of the mechanical exfoliated graphene under the same test conditions. And the value is 3761 cm^2^ V^–1^ s^–1^, as shown in Figure S8c,d of the Supporting Information.

To summarize briefly, we first achieved ultrafast and highly controllable transfer of graphene and put forward a brand new transfer mode based on the original substrate at a melted state. All the characterizations above show that the sliding transferred films exhibit good crystalline integrity and long‐distance continuity without cracks, wrinkles or any smudges. The novel sliding transfer method totally inherits the simplicity and speedability of traditional mechanical cleavage and improves its controllability for uniformity and size. The introduction of rolling–sliding process ensures the large‐area transfer of continuous and uniform graphene, which is an extremely large improvement as compared to traditional mechanical transfer. The selection of liquid metals enables one to obtain high‐quality and strictly uniform monolayer graphene due to its self‐limited catalytic property and the subsequent efficient sliding transfer is resulted from the large delocalization of metallic atoms when in a molten state. In addition, the moderate horizontal sliding force enables the ultracontact and enhanced interaction between graphene and target substrates, which makes it possible to obtain wafer‐size graphene without any cracks, wrinkles, and contaminations. More importantly, this sliding transfer is not strictly limited to the type of the catalytic support or the target substrate. This strategy is also not limited to graphene, and it can be extended to transfer other 2D materials, such as h‐BN. Lower‐temperature sliding transfer via modulating the component of the support and the adherence to various substrates, such as flexible substrates, transparent substrates, and insulated substrates, have been demonstrated and need further exploration for industrial applications in the future.

## Experimental Section

*The Design of Graphene/Ga Layers*: Owing to the good catalytic activity of Ga for catalyzing graphene growth, we achieved such a unique graphene/Ga layers. First, the Ga pellet was put on the W foil under 30 °C. Following, chemical vapor deposition (CVD) growth of graphene is carried out as follows: Ar and H_2_ are introduced at a gas flow rate of 300 and 30 sccm, respectively, with the Ga–W substrate heated to 1020 °C. Next, CH_4_ (10 sccm) is admitted into the chamber for 50 min to initiate graphene growth. After growth, the sample is allowed to cool down in Ar and H_2_ atmosphere which is the same as the heating process until the temperature is close to the room temperature. Figure S1a of the Supporting Information illustrates the formation of graphene/Ga layers, which includes: (1) heating process, (2) growth process, and (3) cooling process.

*General Graphene Transfer Methodology*: The process of transferring the graphene to the target substrates involves spin‐coating PMMA film onto the graphene‐grown substrate and releasing the PMMA/graphene film by etching out Ga film in a diluted hydrogen chloride (1:4) for 1 h. This was followed by a rinse in ultrapure water to remove the metal ions. The PMMA layer was dissolved with hot acetone after the PMMA/graphene film was transferred onto a SiO_2_/Si.

*New Sliding Transfer Methodology with Accurate Control*: The novel sliding transfer strategy is schematically illustrated in Figure S3 of the Supporting Information, where graphene/Ga and a SiO_2_/Si substrate were fixed on the equipment in a face‐to‐face state and contacted with each other by rotating the Z axis controller to a certain value to adjust the contact, and subsequently the two substrates were separated in the horizontal direction to achieve sliding by rotating the X axis controller to a certain value. Finally, the graphene film floating on liquid Ga was separated from the liquid bulk and attached to the SiO_2_/Si substrate with accurate control both in the spatial displacement and the exerted sliding force. This sliding step only takes several seconds. After the sliding exfoliation process, a trace of Ga was left on the graphene surface, which was rinsed thoroughly by the hydrochloric acid.

*Characterization*: Optical images were taken with an optical microscope (Olympus DX51), and Raman spectroscopy was performed with a laser micro‐Raman spectrometer (Renishaw in Via, 532 nm excitation wavelength). The AFM images were taken with a NT‐MDT Ntegra Spectra with graphene transferred onto the 300 nm SiO_2_/Si. The TEM images were taken with an aberration‐corrected high‐resolution TEM (AC‐HRTEM, FEI Titan) operating at 80 kV with the graphene samples directly sliding transferred onto a lacey carbon copper TEM grid. XPS was performed on ESCALAB 250Xi using monochromatic Al Kα radiation (225 W, 15 mA, 15 kV). The images were obtained by a SEM (Zeiss Merlin Compact). Optical absorbance spectrum was collected at room temperature using UV‐6 100PC double beam spectrophotometer (Mapada). The current (*I*)–voltage (*V*) data were collected in a probe station (Keithley 4200) under ambient conditions.

## Supporting information

As a service to our authors and readers, this journal provides supporting information supplied by the authors. Such materials are peer reviewed and may be re‐organized for online delivery, but are not copy‐edited or typeset. Technical support issues arising from supporting information (other than missing files) should be addressed to the authors.

Supplementary
