# Supplementary material for: Controllable Sliding Transfer of Wafer‐Size Graphene
Source: Adv Sci (Weinh). 2016 Mar 15;3(9):1600006. doi: 10.1002/advs.201600006 (PMC5039975; doi:10.1002/advs.201600006)
Supplement: Supplementary file 1 — Supplementary [file ADVS-3-0i-s001.pdf]

Copyright WILEY-VCH Verlag GmbH & Co. KGaA, 69469 Weinheim, Germany, 2016.

ADVANCED SCIENCE
Open Access

Supporting Information

for *Adv. Sci.*, DOI: 10.1002/advs.201600006

Controllable Sliding Transfer of Wafer-Size Graphene

*Wenjing Lu, Mengqi Zeng, Xuesong Li, Jiao Wang, Lifang Tan, Miaomiao Shao, Jiangli Han, Sheng Wang, Shuanglin Yue, Tao Zhang, Xuebo Hu, Rafael G. Mendes, Mark H. Rümmeli, Lianmao Peng, Zhongfan Liu, and Lei Fu**

Supporting Information

Controllable sliding transfer of wafer-size graphene

*Wenjing Lu[§], Mengqi Zeng[§], Xuesong Li, Jiao Wang, Lifang Tan, Miaomiao Shao, Jiangli Han, Sheng Wang, Shuanglin Yue, Tao Zhang, Xuebo Hu, Rafael G. Mendes, Mark H. Rümmeli, Lianmao Peng, Zhongfan Liu and Lei Fu**

Prof. L. Fu, W. J. Lu, M. Q. Zeng, J. Wang, L. F. Tan, M. M. Shao, J. L. Han, T. Zhang, X. B. Hu
College of Chemistry and Molecular Science, Wuhan University, Wuhan 430072, P. R. China
E-mail: leifu@whu.edu.cn
Prof. X. S. Li
State Key Laboratory of Electronic Thin Films and Integrated Devices, University of Electronic Science and Technology of China, Chengdu 610054, P. R. China
Prof. L. M. Peng, S. Wang, S. L. Yue
Department of Electronics, Peking University, Beijing 100871, P. R. China
Prof. M. H. Rümmeli, R. G. Mendes
IFW Dresden, P.O. Box 270116, Dresden 01069, Germany
Prof. Z. F. Liu
Center for Nanochemistry, College of Chemistry and Molecular Engineering, Peking University, Beijing 100871, P. R. China

[§]These authors contributed equally to this work.

1. The inspiration of sliding transfer

Our sliding transfer method is inspired by the natural phenomenon of a snail secreting mucus. When the snail crawls on the ground, the mucus will be exfoliated and a trail will be left behind due to the fluidity of the mucus. As a consequence, the snail can overcome the weak interaction of the mucus to crawl, as seen in Figure S1a. In a similar fashion, given a unique original support (OS) and an appropriate target substrate (TS), when the OS which bears graphene "crawls" on the TS, the graphene will adhere to the TS due to the easy separation of the OS, as seen in Figure S1b. Thus an OS which owns certain fluidity and appropriate viscidity will facilitate the sliding transfer of graphene.

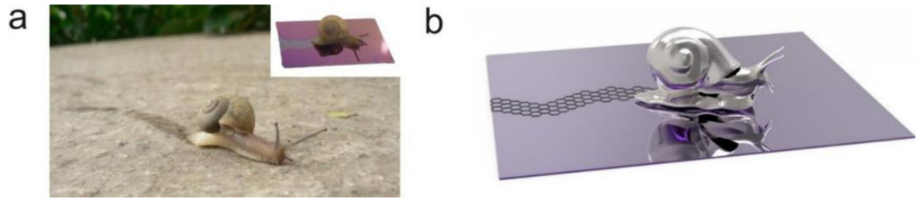

Figure S1. The inspiration of the sliding transfer method for obtaining uniform graphene. (a) Photograph of a real snail crawling on the ground with a trail of mucus left behind and the inset shows the analogous situation on a 300-nm SiO_2/Si substrate. (b) Schematic drawing of a liquid Ga "snail" covered with graphene crawling on an appropriate substrate, with a trail of graphene left behind.

2. The design of 2D materials/Ga layers

I. Pre-treatment

The commercial Ga pellet was divided into small droplets in hot ethanol. A droplet of Ga (20~30 mg) was then placed on a W foil (50 µm thick, cut into 1×1 cm^2). Before loading Ga, the W foils were ultra-sonicated and rinsed with acetone, ethanol and deionized water prior to being dried under a N_2 stream. The Ga pellets with a purity of 99.9999 wt.% and the W foils with a purity of 99.95 wt.% were purchased from Alfa Aesar China (Tianjin) Co. Ltd. and Shanghai Minor Metals Co. Ltd.

II. The design of graphene/Ga layers

Owing to the good catalytic activity of Ga for catalyzing graphene growth, we achieved such unique graphene/Ga layers. First, the Ga pellet was put on the W foil under 30 °C. Following, CVD growth of graphene was carried out as follows: Ar and H_2 were introduced at a gas flow rate of 300 sccm and 30 sccm, respectively, with the Ga–W substrate heated to 1020 °C. Next, CH_4 (10 sccm) was admitted into the chamber for 50 min to initiate graphene growth. After growth, the sample was allowed to cool down in Ar and H_2 atmosphere which was the same as the heating process until the temperature was close to the room temperature. Figure S2a illustrates the formation of graphene/Ga layers, which includes: (1) heating process; (2) growth process and (3) cooling process.

III. The design of h-BN/Ga layers

The method of obtaining the h-BN/Ga layers is similar to that of graphene/Ga layers by CVD. The procedure was as follows: H_2 was introduced at a gas flow rate of 50 sccm, with the Ga–W substrate heated to 1000 °C. Next, B_3H_3 which was heated under 90 °C with a heating mantle was then introduced into the chamber for 30 min to initiate h-BN growth. After growth, the sample was allowed to cool down to ambient temperature in H_2 atmosphere which was the same as the heating process. Figure S2b illustrates the formation of h-BN/Ga layers.

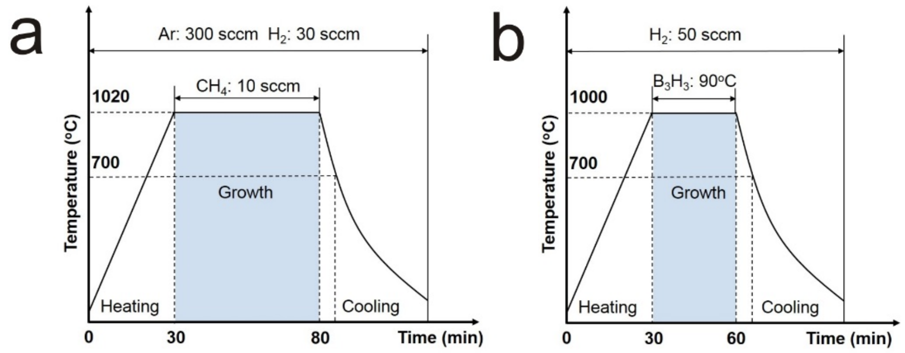

Figure S2. Schematic illustrations of the formation of 2D materials/Ga layers. (a) Procedures for CVD growth of graphene on liquid Ga. (b) Procedures for CVD growth of h-BN on liquid Ga.

3. New sliding transfer methodology with accurate control

The novel sliding transfer strategy is schematically illustrated in Figure S3, where graphene/Ga and a SiO_2/Si substrate were fixed on the equipment in a face-to-face state and contacted with each other by rotating the Z axis controller to a certain value to adjust the contact, and subsequently the two substrates were separated in the horizontal direction to achieve sliding by rotating the X axis controller to a certain value. Finally, the graphene film floating on liquid Ga was separated from the liquid bulk and attached to the SiO_2/Si substrate with accurate control both in the spatial displacement and the exerted sliding force. This sliding step only takes several seconds. After the sliding process, a trace of Ga was left on the graphene surface, which was rinsed thoroughly by the diuted hydrochloric acid. Figure S3 also shows a photograph of an intact graphene film on the SiO_2/Si substrate with a clean surface.

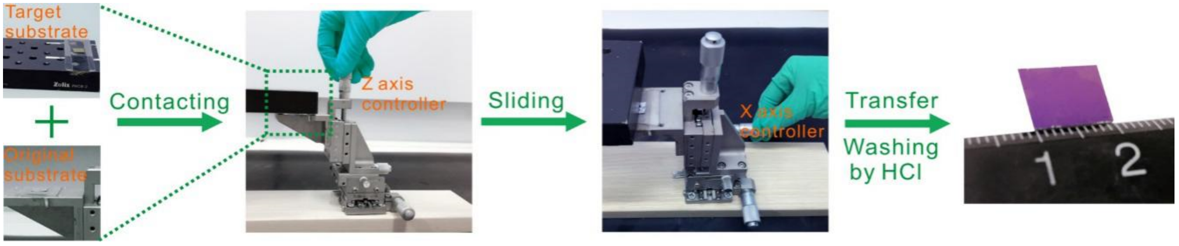

Figure S3. The photos of the sliding transfer process via using an equipment which can achieve an accurate control of contacting and sliding. The photograph of a 1×1 cm^2 monolayer graphene transferred onto a SiO_2/Si substrate through sliding transfer exhibits excellent uniformity and integrality.

4. Sliding transfer of graphene onto other substrates and of other 2D materials onto SiO_2/Si

To highlight the universality of the novel transfer method, the sliding transfer of graphene onto arbitrary substrates and the sliding transfer of other 2D materials were performed. Here, a popular kind of target substrates—transparent quartz and an emerging 2D material—h-BN were adopted as examples. The clean SEM image and Raman spectrum in Figure S4a and c present a defect-free and single-layer graphene on quartz, which are similar to transferred graphene on a SiO_2/Si substrate. Notably, the intensity of the 2D peak at 2677 cm^{-1} with a full width at half maximum (FWHM) of 27.0 cm^{-1} is more than five times the intensity of the G peak at 1582 cm^{-1}. In addition, Figure S4b and d also demonstrate the successful transfer of h-BN by the sliding transfer. The optical image indicates the transferred h-BN film appears to be highly uniform and intact, with no visible defects such as cracks and folds. The Raman spectrum in Figure S4d shows one dominant peak at 1368.7 cm^{-1} which can be assigned to the E_{2g} vibration mode of h-BN and is consistent with the previous reports.[1] These results manifest that the sliding transfer process can be directly extended to other substrates and other 2D materials, which is important for a number of applications of electronic and optical devices, and will open new ground in the field of transferring all two-dimensional materials.

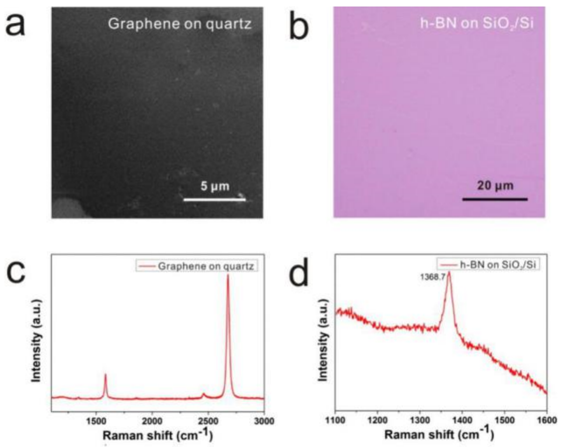

Figure S4. Sliding transfer of graphene onto other target substrates and sliding transfer for h-BN. (a) SEM image of graphene transferred onto quartz, showing a clean surface without contaminations. (b) Optical microscope image of h-BN transferred onto a SiO_2/Si substrate. (c) A typical Raman spectrum of transferred graphene onto quartz. (d) A typical Raman spectrum of h-BN on SiO_2/Si.

5. Lower-temperature sliding transfer: liquid Ga-In and Ga-In-Sn alloys

To achieve lower-temperature sliding transfer, a liquid support which owns lower melting point can be employed. When modulating the ratio of Ga−In alloy to 76%–24% and Ga−In−Sn alloy to 62%–25%–13%, the melting points of these alloys can be as low as 16 °C and 5 °C, respectively, which means that the transfer can be conducted whenever and wherever. As shown in Figure S5a and Figure S5b, clean and uniform graphene films on SiO_2/Si substrates were obtained, in which graphene films were grown on liquid Ga−In and Ga−In−Sn alloys. The typical Raman intensities of D peak of the transferred gaphene are quite small and the intensity ratios of the 2D to G peaks were 1.5 and 1.4, respectively, which show the feature of single-layer graphene.

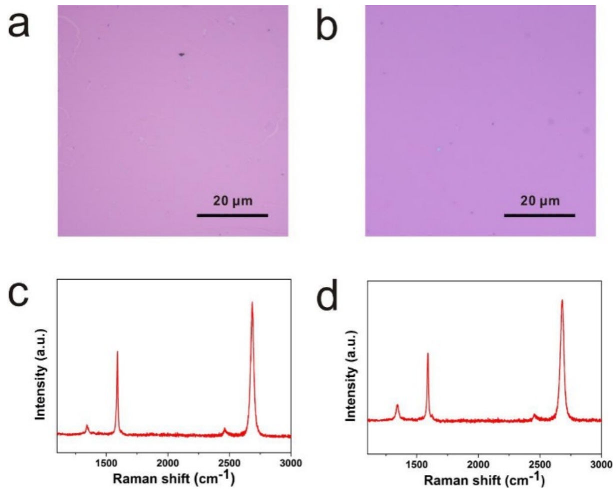

Figure S5. OM images and Raman spectra of graphene transferred from liquid metals with lower melting point. (a) and (b) OM images of graphene transferred from liquid Ga−In and Ga−In−Sn alloys onto SiO_2/Si substrates, respectively. (c) and (d) Typical Raman spectra corresponding to (a) and (b).

6. Comparisons between sliding transferred, mechanical exfoliated and conventional PMMA-transferred graphene on SiO_2/Si

The OM, AFM and edge height statistics comparisons between sliding transfer, mechanical exfoliation and conventional PMMA-transferred graphene on SiO_2/Si are shown in Figure S6. PMMA-transferred graphene on SiO_2/Si is full of PMMA residues and numerous wrinkles, and the edge height is rather large, which is in a range of 0.9~1.2 nm. It is quite apparent that such a PMMA-transferred method is impossible to satisfy the practical applications. For mechanical-exfoliated graphene, graphene size is rather small, and the AFM image presents an inhomogeneous graphene film containing different layer numbers. What is more, the edge height is as large as that of few-layer graphene. These results account for the difficulty in large size and uniformity, and even uncontrollable layer numbers for mechanical-exfoliation graphene. In terms of controllable size and layer numbers as well as relatively reduced residues, sliding transfer can achieve such an ideal target. OM and AFM images of graphene corresponding to sliding transfer are much cleaner, more uniform and free of wrinkles or cracks. Furthermore, the range of the edge height is centered at 0.6 nm, which is comparable to single-layer graphene by mechanical exfoliation. These results above suggest the sliding transfer method a good candidate for researches and applications.

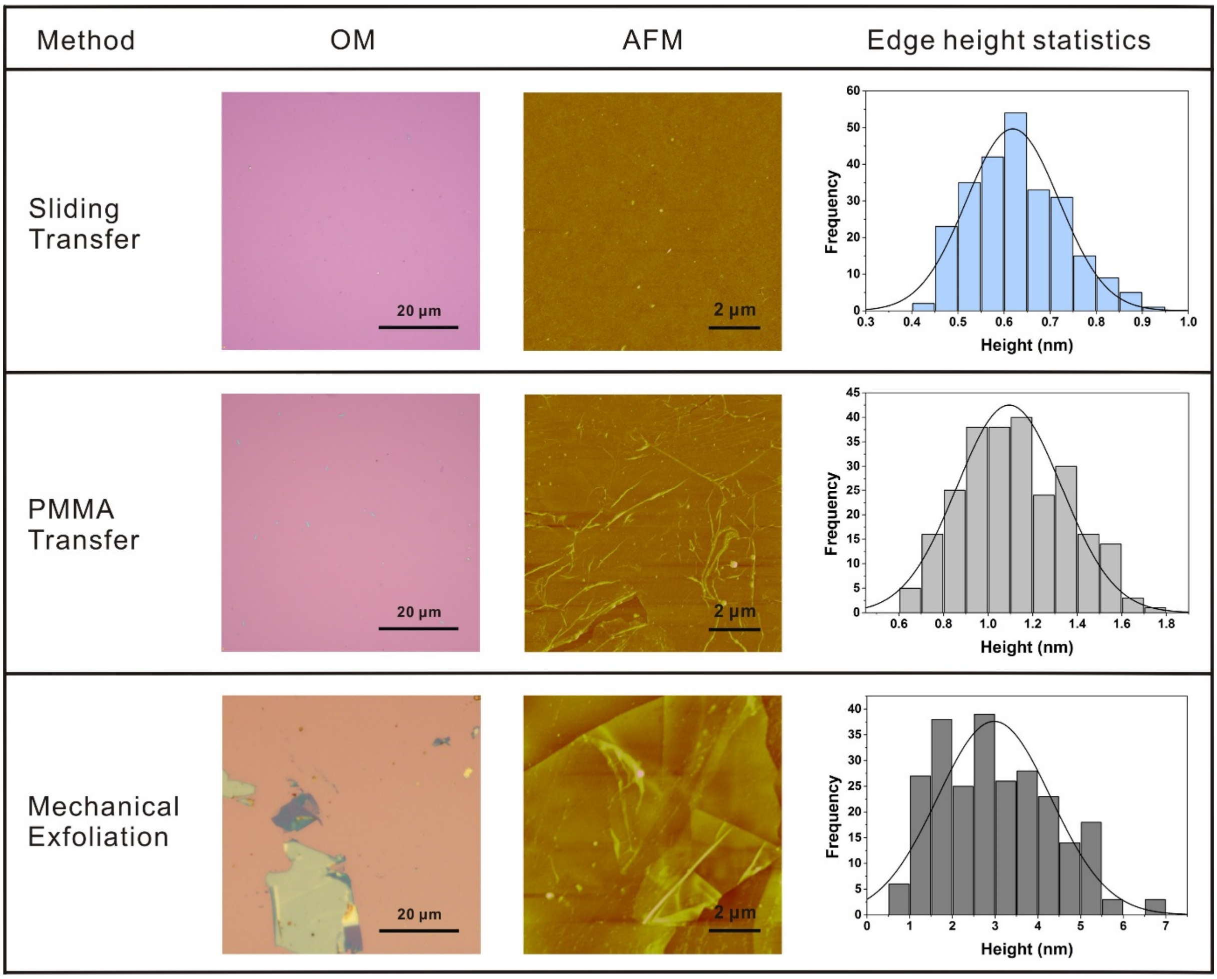

Figure S6. OM, AFM and edge height statistics of sliding transferred, mechanical exfoliated and conventional PMMA-transferred graphene on SiO_2/Si.

7. The rolling-sliding transfer of graphene onto flexible and transparent EVA plastic substrate

Figure S7 shows the schematic illustration of the rolling-sliding process for large-area graphene on EVA plastic. The fast and reliable transfer process involved only a simple step: the roller with a roll of EVA plastic was placed on one end of the liquid Ga plane covered with continuous and large-area graphene (Figure S7a), and was pulled to the other end while the EVA plastic with large-area graphene was pulled up to separate from the original substrate (Figure S7b). Then graphene on EVA plastic with certain amount of Ga (Figure S7c) was washed away by diluted hydrochloric acid (Figure S7d) to remove the Ga within few seconds. Figure S7e shows the photograph of transferred graphene on EVA plastic, which exhibits a large-area, continuous and uniform graphene film. The size of the sliding transferred graphene can be as large as 3 cm × 10 cm.

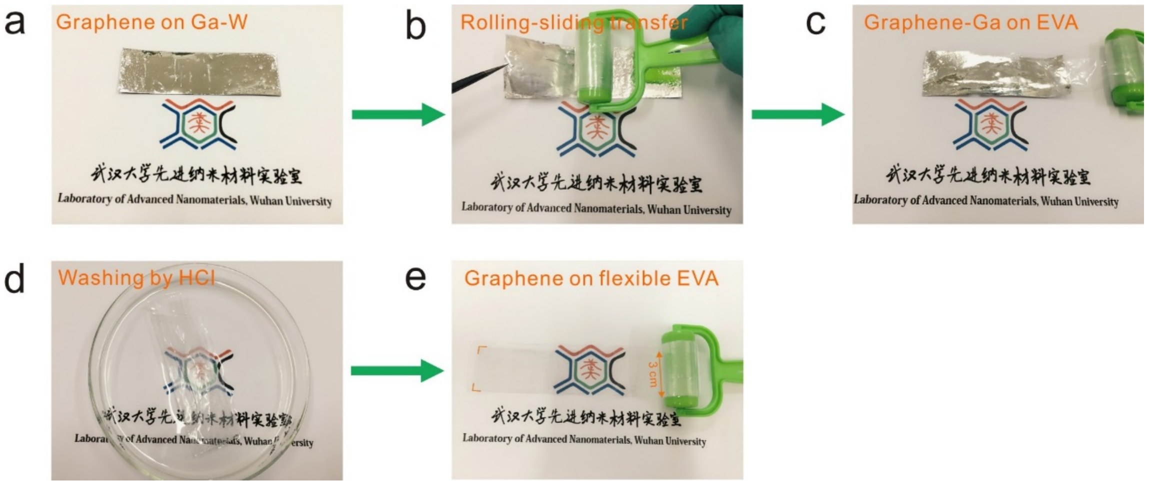

Figure S7. Photograph of rolling-sliding transfer for large-area graphene onto flexible and transparent EVA substrate. (a) The graphene film grown on liquid Ga-W, showing a clean and smooth surface. (b) The rolling-sliding transfer step. (c) Transferred graphene film on EVA plastic with certain amount of Ga. (d) The removal of the Ga via washing by diluted HCl solution. (e) Clean and large-area graphene transferred onto EVA plastic.

8. The application of the flexible, transparent and conductive graphene/EVA film

To show the large-scale, considerably high quality and excellent integrality of the sliding transferred graphene/EVA film, the whole film was used as a transparent conductive film, as seen in Fig 5e. The input voltage was applied to the green LED bulb via the conductive film with commercial wire by DC power supply (DH1720A-5, Beijing Dahua Radio Instrument Co.). Only when the two metal pens simultaneously contacted the transparent film, can the whole circuit be connected, thus the LED bulb would be luminous. With one metal pen fixed on one end of the film and the other pen kept intermittent contact with the other end of the film, the LED bulb would be lightened up on and off, which shows the excellent integrality of the sliding transferred graphene/EVA film.

9. Electrical measurements of graphene via sliding transfer and mechanical exfoliation

The electrical property of sliding transferred graphene was measured through fabricating back-gate field effect transistors on 300-nm SiO_2/Si substrates. As shown in Figure S8a, the source and drain electrodes were deposited using Ti/Pd/Au (0.5/30/20 nm) with a separation of 5 μm. Figure S8b demonstrates transport characteristics (I_{ds}−V_{gs}) of the field effect transistor that was measured under ambient conditions. The extracted carrier mobility of electrons for this device is more than 3625 $cm^2V^{-1}s^{-1}$, which is comparable to the average value of graphene at current conditions. Moreover, we also measured the carrier mobility of the mechanical exfoliated graphene as a comparison, which is 3761 $cm^2V^{-1}s^{-1}$ under the same test conditions, as shown in Figure S8c and S8d. These results demonstrated the high-quality of the sliding transferred graphene.

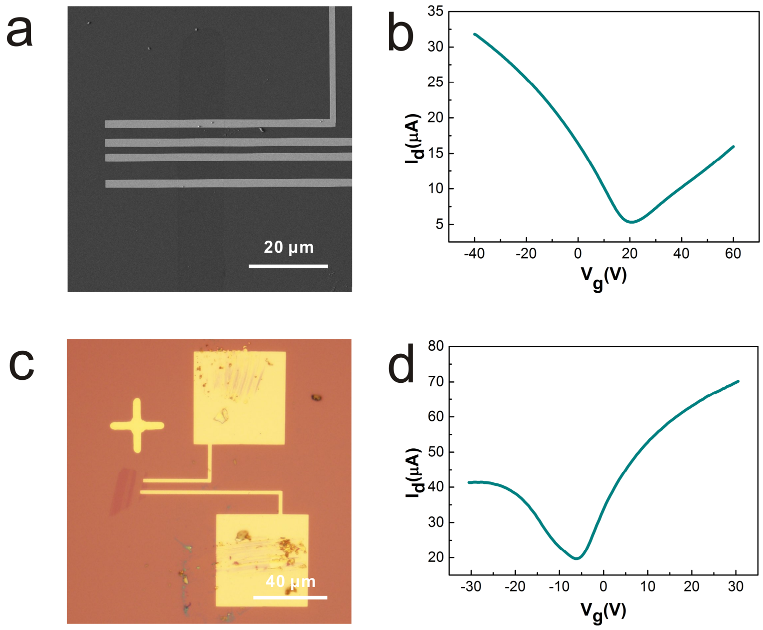

Figure S8. Electrical measurements of graphene samples transferred and exfoliated onto 300 nm SiO_2/Si substrates. (a) SEM image of a back-gate sliding transferred graphene field effect transistor on 300 nm SiO_2/Si substrate. (b) The typical transfer characteristics of the sliding transferred graphene FETs under ambient conditions. (c) Optical image of the mechanically exfoliated graphene FET on 300 nm SiO_2/Si substrate. (d) The typical transfer characteristics of the mechanically exfoliated graphene FETs under ambient conditions.

References

[1] Y. M. Shi, C. Hamsen, X. T. Jia, K. K. Kim, A. Reina, M. Hofmann, A. L. Hsu, K. Zhang, H. N. Li, Z. Y. Juang, M. S. Dresselhaus, L. J. Li, J. Kong, *Nano Lett.* **2010**, *10*, 4134.
